# Effectiveness of Open-Ended Psychotherapy Under Clinically Representative Conditions

**DOI:** 10.3389/fpsyt.2020.00384

**Published:** 2020-05-20

**Authors:** Magnus Nordmo, Nils Martin Sønderland, Odd E. Havik, Dag-Erik Eilertsen, Jon T. Monsen, Ole Andre Solbakken

**Affiliations:** ^1^Department of Psychology, University of Oslo, Oslo, Norway; ^2^Department of Clinical Psychology, University of Bergen, Bergen, Norway

**Keywords:** psychotherapy, representative, effectiveness, outcome, naturalistic

## Abstract

**Objective:**

This study investigates the effectiveness of open-ended psychotherapy in a large, naturalistic, and diverse patient cohort using rigorous and multifaceted assessments.

**Method:**

Patients (N = 370) in open-ended psychotherapy completed an extensive set of self-report measures and diagnostic interviews, including long-term follow-up in order to assess stability of outcomes. About half of the patients qualified for a personality disorder at the onset of treatment. Treatments were open-ended, and on average therapists provided substantially larger treatment doses than common in the literature.

**Results:**

A substantial majority recovered from their respective Axis I (58%) and/or Axis II (55%) disorders during treatment. Patients also experienced large positive changes in self-report measures of overall psychiatric symptoms and moderate positive changes in self-reported interpersonal problems, while very few (< 3%) demonstrated negative development. The patients maintained their diagnostic and self-assessed changes at a two-and-a-half-year follow-up. In contrast, self-reported occupational functioning showed minimal improvement throughout the treatment and follow-up phase.

**Conclusion:**

A naturalistic patient cohort undergoing open-ended psychotherapy demonstrates substantial and stable improvements.

## Introduction

A considerable research effort has gone into the empirical investigation of the effects of psychotherapy. The goal of this research effort is to formulate accurate knowledge, which can inform health-policy and the practice of psychotherapy in general. One major challenge is to navigate the need for stringent protocols to increase internal validity on the one hand, with the need for “representative” research that can generalize to those institutions where the majority of psychotherapy takes place (1). Studies that seek to assess a treatment under real world conditions are typically labelled as effectivness trials, in contrast to lab driven efficacy trials. The distinction between efficacy and effectiveness research is somewhat nebulous as the two terms lack operationalized definitions. Prominent parameters separating the two include the selection of participants, the use of treatment manuals, managing the “dose” of psychotherapy, and the type and amount of therapist training and monitoring (2). Researchers have demonstrated that these procedural variables can influence outcomes in a range of interventions (3). One dramatic example was when Weisz, Weiss & Donenberg (4) concluded that “most clinic studies have not shown significant effects” (p. 1578) after reviewing effectiveness psychotherapy interventions for children and adolescents. The authors contrasted this finding with evidence that efficacy trials from the lab consistently produced significant benefits. Also, what we consider “representative” is a moving target as in that the treatments delivered in routine practice change over time and across nationalities and regions.

There are arguably three main methodologies for assessing representative psychotherapy interventions for adults (5). One is to compare evidence-based treatments (EBT) with treatment as usual (TAU) condition (6, 7). If TAU is found to be equally effective, then this is indirect evidence that the TAU is itself effective. Wampold et al. (7) used meta-analysis to assess EBT *versus* TAU in 14 studies and found that EBT generally outperformed TAU, but that this difference was most likely an effect of heterogeneity in the TAU category, which also included minimal or no treatment. The superiority of EBT disappeared when the authors compared EBT with TAU conditions that contained psychotherapy (three studies). In contrast, Budge et al. (6) found that EBT was significantly more effective in treating personality disorders when compared to a TAU psychotherapy condition, although the amount and type of supervision, therapist training, and therapy dose were not balanced across the conditions (8).

The second line of research comes from direct benchmarking studies that make statistical comparisons between the results from efficacy and effectiveness trials (9). The evidence from benchmarking studies suggests that routine-care does indeed produce similar results compared to the lab (10–12). However, these results are most commonly from patient samples with an unknown degree of pathology with limited or no diagnostic assessments. Most studies only supply description of the primary symptomatic problem. This raises the question of whether patient characteristics such as severity of pathology or the presence of characterological problems might influence the relationship between trial representativeness and outcome. Lastly, the majority of data from benchmarking trials utilize treatment data from American university counseling centers (13). Thusly, these results might not generalize to non-university clinical settings.

The third line of research uses meta-analytical tools to investigate representativeness. Shadish et al. (14) categorized studies with a criterion-based approach. To pass the first criterion the studies had to be conducted in a non-university setting, with patients that were referred *via* traditional routes and used professionals with regular caseloads. To pass the second criterion the studies had to pass criterion one and not use a treatment manual or any monitoring of intervention implementation. To pass the third criterion the study had to pass criterion two, use clients that were heterogeneous with respect to presenting problems, personal characteristics and lastly, not include any explicit therapist training immediately before the study. From 1,082 possible studies that the authors included in the initial pool of meta-analyses, only 56 (5.2%) passed criterion one, 15 (1.4%) studies, passed criterion two and only one study (0.1%) passed criterion three. That study was a family therapy intervention for children (n = 11) with behavioral problems (15). Shadish et al. (14) concluded that criterion one-studies appear to produce similar effect sizes compared with the total sample, but that the lack of criterion two and three studies prohibits strong conclusions regarding the effect of routine-care *versus* lab trials. In a follow-up study (3), the authors expanded the base pool of studies and refined the criteria for representativeness. In place of the earlier stage system, the authors took a dimensional approach, coding the degree of representativeness on a scale from zero to ten. With an expanded pool of baseline studies, including a set of highly representative “clinic therapy” (p. 513) studies, as well as a non-representative set of randomized controlled trials, the authors found that the effects of psychotherapy were robust across the spectrum. A regression analysis of the clinical representativeness features indicated that a large therapy dose, the presence of an internal control group, homogeneity in presenting problem, use of structured therapy, and flexibility in the number of sessions were positively related to effect sizes.

In more recent years, many large-scale randomized pragmatic trials have been conducted, where the aim is to assess the effectiveness of a specific EBT in routine care. These generally support the effectiveness of EBT implemented in routine care (5). A predicament of the pragmatic trial is that it has to balance the need for external validity with the necessity of treatment fidelity. In practice, this means selecting or training therapist to deliver a particular treatment. The very features that the pragmatic trials seek to achieve, namely experimental control, make generalizations to non-EBT clinical settings precarious, although less so compared to the classical lab-driven RCT. The problem of generalization is highlighted by evidence suggesting that therapists seldom implement EBT in their routine care (16). On a related note, evidence from psychopharmacological research suggests that effectiveness trials demonstrate lower effects when compared to their experimental RCT counterparts (17).

Based on this summary of psychotherapy studies under representative conditions it seems that the majority of evidence comes from either a synthesis of heterogenic meta-analytic investigations or from pragmatic trials and benchmarking trials that assess a specific EBT. This synthesis suggests that psychotherapy, on the whole, is effective when implemented under routine-care conditions, but when limiting analyses to highly representative studies, the evidence is limited. Studies have typically severely restricted the number of sessions and rarely provided diagnostic information beyond symptomatic assessment. These characteristics constitute a challenge as the severity of psychopathology, and the presence of personality disorder represent confounding variables. Similarly, “real-world” effectiveness investigations are almost exclusively from university counseling centers. Our goal in this study is to assess the effectiveness of open-ended psychotherapy in a representative healthcare setting with a large, heterogeneous sample, including severe characterological psychopathology. We believe that these results may serve to supplement a literature that is dominated by milder varieties of mental problems. Assessments include rigorous diagnostic interviews as well as patient's self-assessments, both with a considerable follow-up period beyond treatment termination. To our knowledge, the effectiveness of psychotherapy with a representative patient sample, which does not asses a particular EBT, has not been previously documented in the psychotherapy literature. In the present paper we answer the following research questions:

Given an open-ended, naturalistic, and representative psychotherapy setting,

1. What are the rates and magnitudes of diagnostic, symptomatic, and interpersonal change?
2. To what degree are therapeutic gains maintained over time?
3. Do patients experience a positive change in occupational status?

## Methods

### Study Overview

We adopted treatment data collected in the Norwegian Multicenter Study of Process and Outcomes in Psychotherapy (NMSPOP). The NMSPOP is a naturalistic study with a total sample of outpatients (N = 370) gathered from eight treatment sites within the Norwegian public health system in the years 1995–2008. The majority of patients (n = 301) were recruited from psychiatric outpatient clinics spread across 17 separate Norwegian clinics. We also gathered data from the Norwegian University of Science and Technology`s student clinic (patient n = 27). Lastly, we gathered data from outpatient clinics with physiotherapists (patient n = 42) undergoing specialization in psychodynamic body therapy for patients with somatoform disorders (18).

At each of the eight sites, trained coordinators (clinical psychologist or psychiatrist) were responsible for recruiting patients and administering the research protocol. We instructed the coordinators to select patients from their local population randomly, but also to ensure that roughly half had a diagnosable personality disorder. We did not apply any formal randomization procedure. The local coordinators also assessed the patients. The coordinators were all experienced clinicians who underwent training using the assessment instruments. The inclusion policy was liberal, with the following exclusion criteria: age less than 20 years, active psychosis, drug/alcohol abuse as the primary problem, need for emergency treatment or hospitalization, and mental retardation (IQ < 70). These criteria are in line with commonly used criteria in the evaluation of patients for individual psychotherapy at outpatient clinics. The Regional Committee for Medical Research Ethics in Eastern Norway approved the study.

After receiving information and signing a written consent, the patients were submitted to a two-step pretreatment assessment. In the first step, patients completed several self-report questionnaires, including among others a sociodemographic inventory, occupational functioning, the Symptom Checklist-90-Revised [SCL-90-R: (19)] and the Inventory of Interpersonal Problems 64 [IIP 64: (20)]. In the second step, patients underwent a structured diagnostic assessment by the coordinator at each site. This assessment comprised of a Structured Clinical Interview (SCID) based on the Diagnostic and Statistical Manual of Mental Disorders 4^th^ edition (21) criteria for Axis I and II disorders. All assessment interviews and therapy sessions were audio recorded. A subset of the SCID I and SCID II interviews were blindly double-coded by an independent professional to assess inter-rater reliability. The patients were assigned to therapists based on availability after the initial assessment. Patients completed self-report questionnaires during treatment after the 3^rd^, 12^th^, and 20^th^ session. Patients completed self-report questionnaires every 20^th^ session following the 20^th^ session for as long as they received therapy. Following treatment completion, the coordinator repeated the diagnostic evaluation with a SCID I and II interview. The self-report questionnaires were also repeated at the posttreatment assessment. While some of the patients completed their postassessments directly after treatment completion (35%), most completed this assessment a few months after treatment completion due to practical issues. The average delay was 9.8 after treatment completion (SD = 25.5, Median = 2.46). The patients were then assessed with SCID interviews by the same coordinator and completed self-report measures six months, one, and two and a half years following the posttreatment assessment. A subsample (n = 17) of patients also had a six year follow-up assessment.

The therapists (n = 88) were mainly experienced clinicians with a mean of 10 years (SD = 6.5) of psychotherapy experience. All therapist also had postgraduate professional training, including a mean of 5.9 years (SD = 4.3) of clinical supervision. Notable exceptions were the physiotherapists (n = 8) and student therapists (n = 27) who received supervision. The mean number of patients per therapist was 5.6 excluding the student therapists where each student saw one patient. Therapists were instructed to provide their usual therapeutic practice.

#### Sample Characteristics

The mean sample age was 35.2 (SD = 9.4) years with a majority of female patients (69.5%). See **Table 1** for a description of pretreatment sample diagnostic status. The mean number of pretreatment SCID II criteria of personality disorder was 12.7 (SD = 5.8). The pretreatment mean symptom score, as measured by the Global Severity Index (GSI), was 1.28 (SD = 0.61), while the mean rating of interpersonal problems (IIP Global) was 1.49 (SD = 0.52). The patients reported that “the problem which you are now seeking treatment for” had lasted on average 11.7 years (SD = 9.75). The sample did not include patients who sought treatment for a primary substance use diagnosis. The Norwegian healthcare system has a separate subdivision with clinics specializing in the treatment of primary substance use disorders.

**Table 1:** Changes in occupational status and diagnosis frequency.

	Number and percentages of patients		
	Pretreatment*	End of treatment**	Two-year follow-up***
Functioning	202 (55 %)	205 (65 %)	190 (65 %)
Non-functioning	154 (42 %)	110 (35 %)	104 (35 %)
SCID1 Diagnosis			
Presence of SCID 1 diagnosis	321 (87 %)	119 (40 %)	99 (38 %)
Affective disorders	150	56	42
Anxiety disorders	406	109	84
Somatoform disorders	117	26	23
Eating disorders	31	10	4
Substance-Related disorder	9	4	4
Schizophrenia and other psychotic related disorders	2	3	2
SCID2 Diagnosis			
Presence of SCID 2 diagnosis	200 (54 %)	84 (28 %)	58 (22 %)
Cluster A	82	31	14
Cluster B	71	24	21
Cluster C	169	62	35
Not Otherwise Specified	2	0	1

*Percentage from total sample (N = 370).

**Percentage from available occupational (n = 315) and diagnostic (n = 297) post-treatment data.

***Percentage from available occupational (n = 294) and diagnostic (n = 258) follow-up data.

When assessed pretreatment, a subgroup of patients indicated that they used prescribed medication to treat their psychological problems either “regularly” (22%) or “when in need” (7%). The majority of patients using psychotropic medication indicated that they mainly used an antidepressant (n = 72), while fewer indicated that they mainly used an anxiolytic (n = 19), a hypnotic (n = 3), an antipsychotic (n = 4), or pain medication (n = 8). Of the psychotropic medication users, the majority used a single medication (n = 70), while some were prescribed two (n = 22), three (n = 9) or four (n = 4) different medications.

### Assessment Instruments

#### The Symptom Checklist 90 Revised (SLC-90)

We used the SLC-90 to assess overall symptom presence and severity. It contains 90 questions asking patients to rate, on a Likert scale from 0 (not at all) to 4 (very much), the intensity of a given symptom during the last week. The symptoms represent nine dimensions of distress, which can be further grouped into three global indexes (22). We used the Global Severity Index (GSI) as an overall symptom severity measure, which is the mean rating across the entire checklist. The GSI is a robust measure of overall symptom severity (23). The SCL-90 demonstrated high internal validity in our sample with a pretreatment Cronbach's alpha of.97.

#### The Inventory of Interpersonal Problems 64 (IIP-64)

We applied the IIP-64 to assess levels of interpersonal problems. It consists of 64 questions rated on a five-point Likert scale from 0 (*not at all*) to 4 (*very much*). The first 39 questions begin with the phrase “*It is hard for me to…*” while the remaining 25 questions ask about “*Things that I do too much*.” We used the IIP global, which is the mean scores across the entire inventory. This global score has been shown to adequately capture a wide range of interpersonal problems and pathology (24). The IIP-64 demonstrated high internal validity with a pretreatment Cronbach's alpha of.93.

#### Occupational Status

We created a dichotomous variable (occupational functioning vs. no functioning) based on self-reported occupational status. We classified the following responses as “functioning”: 1) “I am currently engaged in paid work,” 2) “I am a stay-at-home mom/dad,” 3) “I am currently engaged as a student” or 4) “I am retired.” We classified “non-functioning” with the following responses: 1) “I am currently on sick leave,” 2) “I am currently in work rehabilitation,” 3) “I am currently receiving disability benefit” or 4) “I am currently unemployed.” We assessed changes in occupational status by comparing pre- to posttreatment levels and pre- to follow-up measurements. We used the last recorded assessment in our follow up assessment which was six years for 4.9% (n = 17) of the sample, two and a half years for 68.6% (n = 254), one year for 5.1% (n = 19) and six months for 1.0% (n = 4). A total of 20.5% (n = 76) and 14.9% (n = 55) had no follow-up measurement of occupational status at follow-up and posttreatment respectively.

#### SCID Interview

The SCID interview was developed for the assessment of both Axis I clinical disorders (SCID I) and Axis II personality disorders (SCID II) according to the Diagnostic and Statistical Manual of Mental Disorders (21). Each disorder is associated with a set of items that assess different manifestations of the disorder. Each item can be scored as either absent, sub-threshold, true, or “inadequate information to code.” The items corresponding to each particular disorder is then summed to assess whether the patient qualifies for a given disorder. The SCID interviews have been shown to give reliable assessments of DSM-IV diagnoses (25). To assess changes in diagnostic status for each Axis, we employ a dichotomy between no diagnosis (0) and one or more diagnoses present (1).

A sample of 40 SCID I and 20 SCID II interviews were selected at random to assess inter-rater reliability. We found that Cohen's Kappa for Axis I disorders ranged from.53 to 1.00 with a mean of.75, indicating fair too excellent agreement. Cohen's Kappa for Axis II disorders ranged from.63 to 1.00 with a mean of.82, indicating good to excellent agreement.

### Statistical Analysis

We carried out all statistical analyses using R version 3.5.2 (26). We used the lme4 package (27) to fit linear mixed models, and lmerTest (28) equipped with Satterthwaite's degrees of freedom method for p-values. We used ggplot2 (29) and ggalluvial (30) to make the figures. Imputation was performed using the mice package (31).

#### Longitudinal Self-Report Questionnaires

We analyzed the data using Multilevel Modeling (MLM) with Bayesian Information Criteria (BIC) as our indicator of model fit. MLM is the recommended method for analyzing longitudinal and repeated health measures as it allows the nesting of each measurement (level 1) within each patient (level 2). MLM has been shown to outperform traditional methods (*e.g.*, last observation carried forward method) when handling missing data and accounting for potential dropout bias (32, 33). For our treatment phase model, we applied the session number as a fixed occasion time estimate, as this allows for between-subjects comparisons of regression coefficients. We centered treatment start at zero and coded the posttreatment assessment as the last session number from the longest treatment series, plus 1, which was 361. For our follow-up model, we centered time as zero at treatment completion and used month after treatment completion as our time measure.

We began our analysis by visually inspecting GSI and IIP-Global raw scores. We observed a log-linear distribution for the majority of cases on both outcome measures, both in the treatment- and follow up phase. To assess this, we ran models with both linear time, where each time-point corresponds to the specific time of measurement, and with log-transformed time, where each time-point is multiplied by the log^10^ to produce a log-linear curve. These analyses confirmed the superior fit of log-linear slopes on both our main self-report outcome measures with lower BIC values as compared to linear time models. The superior fit of log-time was true for both the treatment phase and the follow-up phase, although the rate of change was marginal during follow-up. Another benefit that is of particular importance in our open-ended design is that log-linear time places the last observation (*e.g.*, the postmeasurement) closer to its original time-point, thereby lessening any potential skew produced coding post time as 361 on patients with short treatments (34).

Several investigations have shown that latent therapist effects can influence the statistical modeling of patient trajectories (35–37). Therefore, in addition to the analyses presented below, we also carried out a separate three-level (measurements, patients, therapist) model. This model produced similar overall results and a poorer BIC value compared to the two-level model (measurements, patients). We also performed a separate analysis with patients nested within treatment site, also with very similar results and worse BIC value, leading us to omit these results.

#### Effect Sizes and Clinically Significant Change

We used Cohen's *d* to estimate the magnitude of change by dividing the estimated change score with the corresponding pooled standard deviation. The pooled standard deviation was estimated by taking the mean standard deviation from all measurement points with more than 150 patients, and merging the treatment phase and follow-up phase into a single measure. The pooled standard deviations were 0.56 for GSI and 0.51 for IIP Global. Our post measurement standard deviations were counted twice as both the end of the treatment phase, and as the start of the follow-up phase, corresponding to our two multi-level models. Using a pooled standard deviation reduces the problem of artificially inflating the effect size (38). We applied Cohen's (39) standard for categorizing effect sizes, where 0.2–0.5 is a small effect size, 0.5–0.8 is a moderate effect size, and > .08 is a large effect size. Clinically significant change was calculated according to Jacobsen and Truax's (40) definition in which a patient's predicted score needs to achieve both a reliable statistical change (41) and pass the cut-off to a functional population as compared to a dysfunctional population. We used the Norwegian norms from Carrozzino et al. (42) and Monsen, Hagtvet, Havik, and Eilertsen (43) and calculated these cut-offs to be 0.73 and 1.22 for the GSI and IIP Global, respectively. We defined a patient as recovered if he or she met both criteria. We further categorized patients as reliably improved if they demonstrated a statistically reliable change, but failed to cross over the cut-off of dysfunction; unchanged, if they did not demonstrate a statistically reliable change; or deteriorated, if a patient showed a negative, statistically reliable change. We calculated these criteria for both of our main outcome measures at the end of treatment and the final follow-up assessment.

#### Diagnostic and Occupational Status

Using the exact2x2 R package (44), we applied two-sided McNemar tests with continuity corrections and odds-ratio tests to analyze changes in diagnostic and occupational status from pre- to posttreatment, and pretreatment to follow-up status. This test obtains its respective p-values through central hypergeometric distribution (45). Both measures were coded as either 0 (No diagnosis/Functioning) or 1 (Diagnosis/Non-functioning).

#### Registration

To increase transparency, we registered our hypotheses and statistical analyses using the Open Science Framework (46) before running our analyses. We completed this registration after the data had been collected and preliminary analyses had been conducted. The results of the preliminary analyses were presented at the 39th International Meeting of the Society for Psychotherapy Research conference. We have made deliberate efforts to prevent the preliminary analyses from affecting our current results by not subdividing our data, including all relevant outcomes measurements and selecting standard statistical tools for multilevel longitudinal data.

## Results

### Patient Flow and Data Completeness

**Figure 1:**
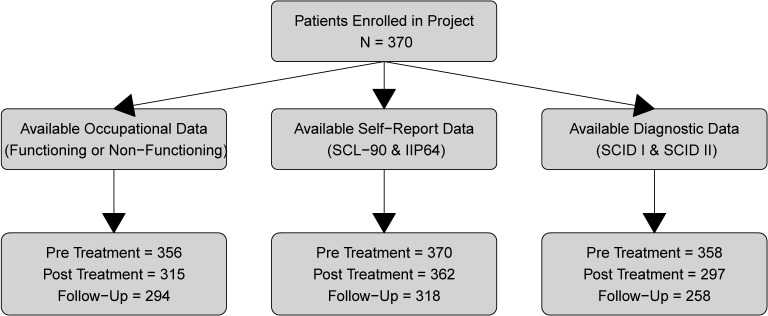
Patient attrition.

Out of the original sample of 370 patients, eight did not start treatment or did not give any assessment following the pretreatment assessment, giving a total treatment sample of 362. When analyzing diagnostic and occupational follow-up data, the last available assessment was used. A few patients had a last measurement of six years (n = 23) after treatment, while the majority of the patients had follow-up assessments approximately two and a half years after treatment completion (Mean = 28.1 months, SD = 8.6). A total of 52 (14.1%) patients had no follow-up measurement of main outcomes. Logistical regression was performed on both the GSI and IIP using pre- and posttreatment scores to predict missing follow-up data. Patients' pre (*p* = 0.79) and post (*p* = 0.55) treatment GSI scores did not predict missing GSI at follow-up. However, both pre-, χ2(1) = 1.32, *p* = 0.014, and posttreatment, χ2(1) = −1.13, *p* = 0.027 IIP Global scores were statistically significant predictors for missing IIP Global at follow-up. The multi-level models were fitted with all available data. The data collection of self-report, diagnostic and occupational status represents distinct and separate processes and therefore have distinct attrition patterns. See **Figure 1** for a complete description of patient attrition.

**Figure 2:**
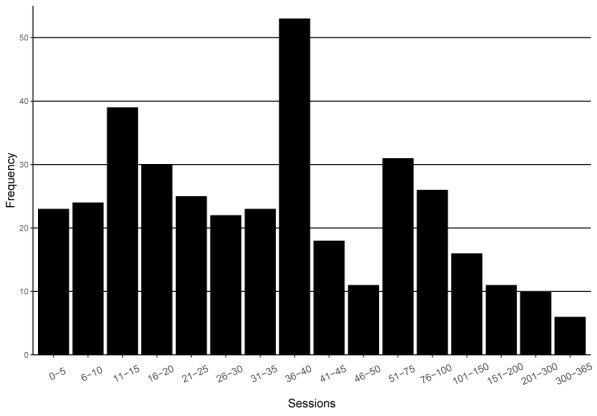
Treatment length.

The distribution of therapy length is shown in **Figure 2**. The mean number of sessions was 51.3 (SD = 58.9) with a median of 35. A few patients with very long therapies accounted for the high variance. Every treatment was terminated by a joint agreement between the therapist and the patient except for therapy dropouts with one exception: One of the sites (therapist n = 7, patient n = 31) had an upper-limit of 40 sessions.

### Diagnostic and Occupational Status

**Table 1** shows changes in diagnostic and occupational status. We apply the terminology that positive change equals fewer diagnoses. We found a positive statistically significant difference of any symptom diagnosis between pre- to posttreatment, *x*^2^(1, n = 293) = 128.8, *p* < 0.01, OR = 19.1, 95% CIs [9.5, 45.1]. We also found a positive statistically significant change when comparing presence of any symptom diagnosis pretreatment to follow-up, *x*^2^(1, n = 254) = 109.5, *p* < .01, OR = 15.1, 95% CIs [7.7, 33.8]. When applying the same test with the presence of any personality disorder diagnosis we found the positive changes from pre- to posttreatment to be statistically significant, *x*^2^(1, n = 294) = 59.2, *p* < .01, OR = 8.1, 95% CIs [4.3, 16.8]. Lastly we found a positive statistically significant change in presence of any personality disorder from pretreatment to follow-up status, *x*^2^(1, n = 257) = 65.7, *p* < .01, OR = 8.2, 95% CIs [4.5, 16.3].

**Figure 3:**
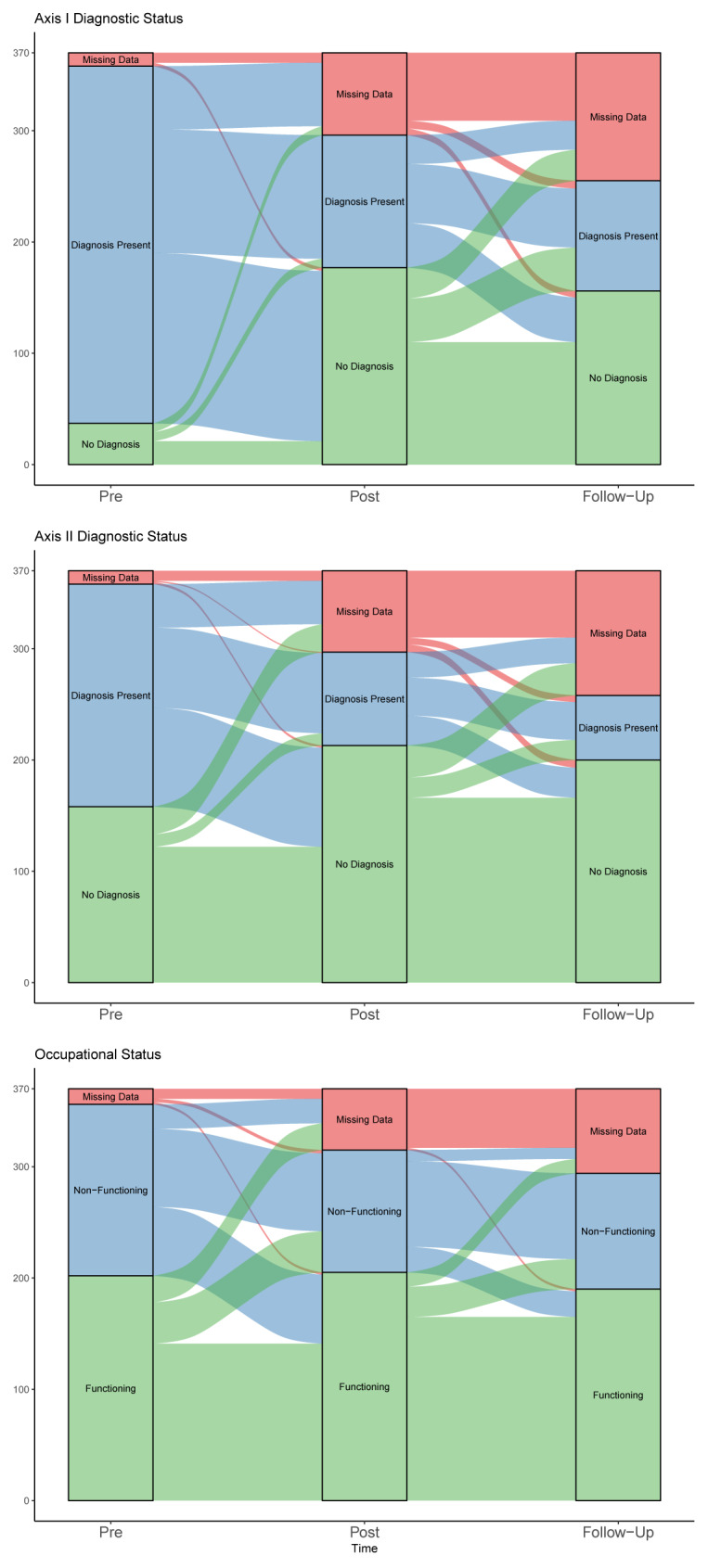
Alluvial diagram of changes in diagnostic and occupational status.

We found that overall, changes in self-reported occupational status were minimal through the treatment and follow-up phase. The majority of patients kept their respective status through the treatment and the follow-up phase, while a substantial minority went from non-functioning to functioning. Relatively few patients had a negative development from functioning to non-functioning. See **Figure 3** for an alluvial development diagram. A McNemar test revealed a statistically significant positive change in occupational functioning from pre- to posttreatment, *x*^2^(1, n = 310) = 5.8, *p* = 0.12, OR = 1.68, 95% CIs [1.1, 2.6], indicating that more people were able to work posttreatment, compared to pretreatment. The trend was maintained when we compared pretreatment to follow-up occupational status, *x*^2^(1, n = 291) = 2.4, *p* =.12, OR = 1.38, 95% CIs [0.9, 2.1].

**Figure 4:**
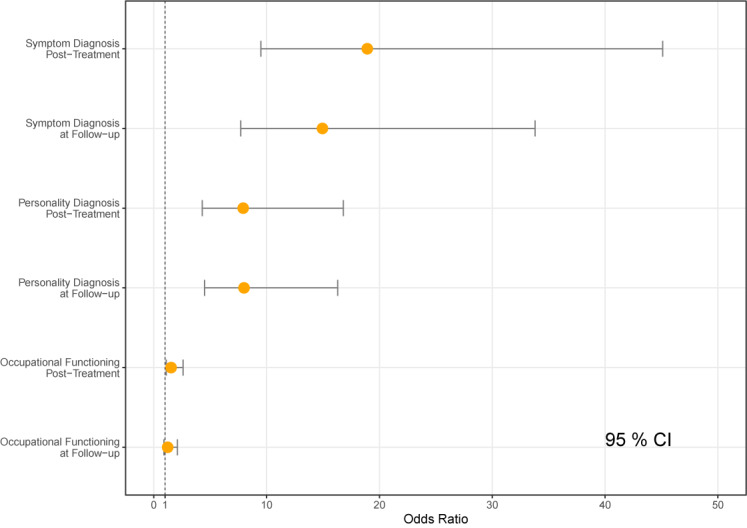
Odds ratios comparison to pretreatment.

To account for missing occupational data we performed a pair of analysis using the Multivariate Imputation by Chained Equation methodology. Missing occupational data were imputed by using available diagnostic and occupational status, gender, GSI, and IIP Global. We created 30 datasets with a maximum of 20 iterations, using parallel socket cluster. When analyzing the complete dataset we found that pre- to posttreatment remained significant, x^2^(1, n^imp^ = 370) = 6.7, p = 0.02, OR = 1.56, 95% CIs [1.1, 2.3], as did pre- to follow-up, x^2^(1, n^imp^ = 370) = 5.48, p =.02, OR = 1.51, 95% CIs [1.1, 2.1]. See **Figure 4** for a graphical visualization of Odds Ratio scores.

### Change in Symptoms and Interpersonal Problems During Treatment

**Figure 5:**
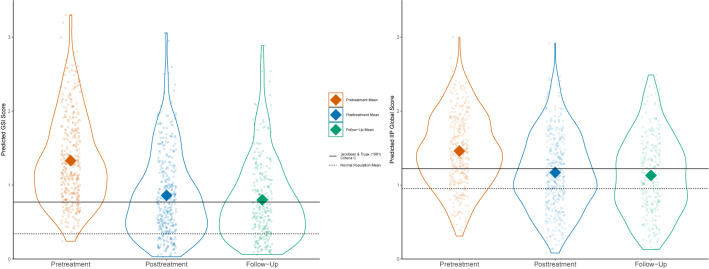
GSI and IIP Global predicted score distributions.

The multilevel models from the main outcome treatment phase are shown in **Table 2**. We found that scores on the GSI were subject to a statistically significant change during the treatment phase when allowing for variable change across patients (Model 1), *β* = −.18, *p* < .01. This was also the case for IIP Global (Model 1), *β* = −.11, *p* < .01. For the GSI, the overall intercept in the treatment phase was estimated to be 1.32. Overall change across the treatment phase was estimated to be a reduction of.47 points. For IIP Global the overall intercept was estimated to be 1.45 with a total treatment change of 0.29. There was substantial heterogeneity across the sample concerning both the GSI and IIP Global as indicated by the significant variance in the intercepts. The significant variance in slopes on both main measures indicates a high degree of variability in treatment response, which emphasizes the need for multilevel analysis. The necessity of multilevel analysis is also indicated by the lower BIC when comparing Model 1, which allows for variable change across patients, with Model 0, which does not. See **Figure 5** for a visualization of predicted scores.

**Table 2:** Measures on symptoms and interpersonal functioning in the treatment phase.

	GSI		IIP Global	
	Model 0	Model 1	Model 0	Model 1
	Est	Est	Est	Est
*Fixed effects*				
Intercept	1.33** (.03)	1.33** (.04)	1.45** (.03)	1.46** (.03)
Logtime	-.186** (.01)	-.186** (.01)	-.108** (.008)	-.113** (.012)
*Random effects*	Est	Est	Est	Est
Residual	.14** (.37)	.10** (.32)	.09** (.30)	.07** (.26)
Variance in intercept	.31** (.56)	.39** (.63)	.24** (.49)	.027** (.02)
Variance in slopes	N/A	.04** (.21)	N/A	.03** (.17)
Intercept Slope Corr.	N/A	-.43	N/A	-.32
BIC	2714.5	2555.8	1952.8	1798.3

Standard error in parenthesis. Estimation performed using Restricted Maximum Likelihood (REML). Model 0 fixates the rate of change for each patient while Model 1 allows for variable changes across patients. Fixed effects are presented with the estimate and standard deviation in parenthesis. Random effects are presented with the variance and standard error in parenthesis. **p < .01.

### Symptoms and Interpersonal Problems in the Follow-Up Phase

The results from the multilevel model of both main outcome variables during the follow-up phase are shown in **Table 3**. The follow-up phase was associated with a small but significant drop in interpersonal problems as measured by the IIP Global when allowing for variable changes across patients, *β* = −.04, *p* < .001. The overall intercept was estimated to be 1.19 with a total reduction of.064. The GSI also show a further reduction, but this change was not significant *β* = −.008, *p* = 0.49. The overall intercept in the follow-up phase was estimated to be 0.81 with a total reduction of.012 points. Overall, this indicates that treatment gains were maintained. The follow-up phase also showed significant variance in both intercepts and slopes.

**Table 3:** Measures on symptoms and interpersonal functioning in the follow-up phase.

	GSI		IIP Global	
	Model 0	Model 1	Model 0	Model 1
	Est	Est	Est	Est
*Fixed effects*				
Intercept	.82** (.04)	.82** (.04)	1.19** (.03)	1.19** (.03)
Logtime	-.009 (.01)	-.008 (.02)	-.042** (.01)	-.043* (.01)
*Random effects*	Est	Est	Est	Est
Residual	.08** (.28)	.07** (.26)	.07** (.26)	.06** (.23)
Variance in intercept	.34** (.58)	.38** (.61)	.28** (.53)	.30** (.55)
Variance in slopes	N/A	.03* (.17)	N/A	.02** (.14)
Intercept Slope Corr.	N/A	-.32	N/A	-.22
BIC	1199.8	1191.7	996.9	995.8

Standard error in parenthesis. Estimation performed using Restricted Maximum Likelihood (REML). Model 0 fixates the rate of change for each patient while Model 1 allows for variable changes across patients. Fixed effects are presented with the estimate and standard deviation in parenthesis. Random effects are presented with the variance and standard error in parenthesis. *p < .05 **p < .01.

### Clinically Significant Change and Effect Sizes

**Table 4** shows clinically significant change at the level of the individual (40). The majority of patients (69%) experienced a reliable improvement or complete recovery in terms of overall psychiatric symptoms as measured by the GSI. This symptomatic improvement was sustained during the follow-up period. For interpersonal problems, about one third (35%) of the patients made either complete recovery or a reliable positive change. The IIP Global revealed fewer complete recoveries compared to the GSI. During the treatment phase, the GSI change was large (*d* = 0.85) while the IIP Global change was moderate (*d* = 0.57). The follow-up phase showed high stability, with the GSI demonstrating a weak improvement (*d* =.03) which was not statistically significant, while the IIP Global demonstrated a somewhat stronger improvement (*d* = 0.13) which was statistically significant.

**Table 4:** Clinically significant change using predicted scores.

	Number and Percentages of Patients	
Measures and status	Pretreatment to termination of treatment *	Pretreatment to two-year follow-up **
Global Severity Index (SCL-90)		
Recovered	142 (38 %)	132 (42 %)
Improved	114 (31%)	72 (23 %)
Unchanged	107 (29 %)	103 (32 %)
Deteriorated	6 (1.6 %)	11 (3.4 %)
IIP Global (IIP-64)		
Recovered	84 (23 %)	99 (31 %)
Improved	43 (12 %)	42 (13 %)
Unchanged	234 (63 %)	167 (53 %)
Deteriorated	9 (2.4 %)	10 (3.1 %)

* Percentage from total sample (N = 370).

** Percentage from patients with follow-up measure (n = 318).

## Discussion

This study shows that patients who receive psychotherapeutic care in an open-ended outpatient format, experience large to moderate positive change on self-report measures of overall psychiatric symptoms and interpersonal difficulties, as well as large observer-rated diagnostic improvements. The majority of patients do not qualify for an Axis I or Axis II diagnosis at the end of treatment and can be categorized as either reliably improved or recovered. Patients experienced a more substantial reduction in general psychiatric symptom compared to interpersonal problems which they several years after treatment termination. During the follow-up phase, the patient's experiences further positive changes in interpersonal functioning, whereas they maintained their level of general psychiatric symptoms. The improvements seen in interpersonal functioning during the follow-up might be a positive consequence of the open-ended nature of treatment, where patients and therapists are free to focus on characterological problems in contrast to manualized, time-limited and symptom-focused interventions.

As this study is a cohort study without a control group, we cannot ascertain a causal link between patient improvement and the psychotherapy received. Due to ethical concerns (47) and practical challenges (48), few studies focus on untreated psychiatric populations. The lack of studies on untreated psychiatric populations makes it difficult to compare our treatment sample with a hypothetical no-treatment control. The studies that do exist are mainly on patients with a mild to moderate mood and/or an anxiety disorders (49, 50). These studies suggest that spontaneous recovery for anxiety and mood disorders is common, but estimates vary greatly between studies. Spontaneous recovery is documented to be rarer for patients diagnosed with a comorbid personality disorder (51–54) than for patients without. There is a lack of data for recovery rates of severely ill populations that are not in treatment as these individuals are typically high-treatment utilizers (55). We would argue that the samples presented in the spontaneous recovery literature is of milder psychopathology compared to our sample, where 54% fulfilled the criteria for one or more personality disorders at admission. The robust positive changes seen in our sample seems greater than what one might expect from the rate of natural recovery found in each disorder. We also believe that the observation that our sample maintained the therapeutic gains during the follow-up is indicative that positive changes should be ascribed the therapy received. This lasting change is contrasted to the chronicity reported pretreatment. Although randomization to a control condition can open the road to causal analyses, we believe that that this is precarious for research on long-term treatments for severely ill patients that seek to compare routine care and spontaneous recovery. In such a scenario, the control condition would have to limit or omit routine care for several years forcefully. This dynamic is also apparent in the controversy surrounding evidence-based therapies (56).

The substantial positive changes in overall symptoms and interpersonal problems did not correspond to an equally substantial positive change in occupational functioning. The changes seen in occupational functioning were negligible. This finding is sharply contrasted by the clinical recovery and improvements observed for the majority of patients. This finding is in line with research that indicates that disability interventions that exclusively focuses on the treatment of a mental disorder rarely produces occupational recovery (57) as they overlook the complex interaction of work perceptions and challenges, attitudes, beliefs and other psycho-social influences (58, 59). Our findings indicate that patients can improve substantially, as measured by self-reported mental health questionnaires and observer-rated psychiatric diagnosis, and still see a meager degree of positive change in occupational status. This result should be interpreted with caution as missing posttreatment and follow-up data obscures the analysis. Another concern is related to our measurement of occupational functioning. Returning to work is a complex phenomenon (60) and a single-item operationalization might lack the sensitivity to detect intricate changes.

The outcome measure with the most substantial overall change was psychiatric diagnoses as measured by the SCID interviews. The majority of patients did not qualify for a diagnosis after the treatment phase. This improvement was largely maintained in the follow-up phase and true for both Axis I and II diagnoses. Previous research has found that clinicians and independent observers usually report more substantial positive change when compared to patients assessing themselves (61, 62). In the present study, an independent coordinator, and not the respective clinician performed the diagnostic interviews and assessment, so the comparison to research on clinician versus patient ratings may be inaccurate. We believe that the independence of the coordinators adds to both the validity and reliability of our diagnostic data.

Using predicted scores, we found a surprisingly low amount of deterioration (1–3%) compared to what is generally found in the adult psychotherapy (5–10%) literature (5). We did not expect low amounts of deterioration as effectiveness interventions are usually associated with higher rates of deterioration when compared to structured efficacy trials (63). We believe that the low rate of deterioration is caused by either of two explanations or a combination of the two: Firstly, the fact that this is an open-ended treatment might have given the therapist the possibility of sustaining treatment until he or she was convinced that the patient was well enough to terminate treatment. This feature is in contrast to treatments with a prescribed set of sessions where a patient might face a setback or drop in functioning at the end of the prescribed amount of sessions, thereby giving the patient a negative skew on his or her predicted slope. Indeed, others have argued that time constraints can have an impact on successful termination (64). With an open-ended format, the patient can continue in therapy until the crises have been overcome and normal functioning has been established, thereby lessening the negative skew effect of the transient crises. Indeed, it has been posited that the experience of control over therapy conditions is directly intertwined with patient improvement (65).

### Limitations and Future Directions

A few limitations should be considered when assessing the degree of representativeness of our procedures and sample. Firstly, we did not randomize the selection of therapist to participate in this trial; rather therapists were self-recruited based on availability at each site. Second, patients were not formally randomized to participate in the trial. Instead, the local administrator of each treatment site was instructed to pick out patients as randomly as possible, while striving to provide a representative sample for his or her respective treatment site. No information was collected from patients who declined the invitation to participate. Our therapist sample was comprised mostly of experienced professionals who volunteered to be a part of an intensive research project. A potential selection effect could have excluded underperforming therapist who might have contributed to a higher rate of deterioration (66).

Another, more substantial question is to what degree this study captures the elements of what is considered “representative” psychotherapy practice. This question is hard to answer as routine care is a moving target that changes with time and across national and regional borders. Examples of this process can be seen in the proposed movement towards evidence-based and stepped-care treatments initiated with the Improved Access To Psychological Therapies (IAPT) reform in the UK (67) and its American (68) and Norwegian (69) equivalent. These are examples of possible healthcare reform that could substantially change what we mean by psychotherapy in routine care. The moving target phenomenon is also evident from the psychotherapy research literature. Virtually no psychotherapy effectiveness research has been published in the last ten years when defining effectiveness as routine care that does *not* prescribe a specific treatment for a specific diagnostic population. Therefore, we believe that our results fill an important missing piece in the current psychotherapy literature. In our opinion, these results make a fair representation of what one could expect from the “classical” way of delivering psychotherapy, where therapists are free to choose the treatment methodology, do not have a preset time-constraint, and treat each patient individually, face-to-face. We plan on completing further moderator analyses in future publications.

## Data Availability Statement

The datasets generated for this study are available on request to the corresponding author.

## Ethics Statement

The studies involving human participants were reviewed and approved by Regional Committees for Medical Research Ethics—South East Norway. The patients/participants provided their written informed consent to participate in this study.

## Author Contributions

The work presented is the result of a cooperative effort from the respective authors. Each author has made significant contributions in the development of this manuscript. This includes, but is not limited too, planning the publication, writing the manuscript, analysing data and formulating theoretical and clinical implications.

## Funding

The present article was funded by grants from: 1. The Norwegian Research Council; Health and Rehabilitation through the Norwegian Council for Mental. 2. The Department of Psychology, University of Oslo.

## Conflict of Interest

The authors declare that the research was conducted in the absence of any commercial or financial relationships that could be construed as a potential conflict of interest.
